# Optimizing the Treatment Schedule of Radiotherapy Combined With Anti-PD-1/PD-L1 Immunotherapy in Metastatic Cancers

**DOI:** 10.3389/fonc.2021.638873

**Published:** 2021-03-30

**Authors:** Yuehong Kong, Yifu Ma, Xiangrong Zhao, Jie Pan, Zhi Xu, Liyuan Zhang

**Affiliations:** ^1^Department of Radiotherapy and Oncology, The Second Affiliated Hospital of Soochow University, Suzhou, China; ^2^Institution of Radiotherapy and Oncology, Soochow University, Suzhou, China; ^3^Suzhou Key Laboratory for Combined Radiotherapy and Immunotherapy of Cancer, Suzhou, China; ^4^Department of Pharmacy, The Second Affiliated Hospital of Soochow University, Suzhou, China; ^5^Department of Medical Affairs, ICON Plc, Beijing, China

**Keywords:** metastatic cancer, PD-1/PD-L1 inhibitor, radiotherapy, *in-situ* tumor vaccination, biological response modifiers

## Abstract

Immune checkpoint inhibitors (ICIs) targeting programmed cell death protein-1 (PD-1), and programmed cell death ligand-1 (PD-L1) have been approved for a variety of malignant tumors and are widely used to treat patients with metastatic disease. However, the efficacy of PD-1 inhibitors is limited due to tumor heterogeneity, high tumor burden, and “cold” tumor microenvironment. Radiotherapy can improve the anti-tumor effects of PD-1/PD-L1 inhibitors in various ways. As a new radiotherapy method, stereotactic body radiotherapy (SBRT) or hypofractionated radiotherapy (HFRT) provides higher doses per fraction to the target lesions, thus achieving immune activation effects and overcoming tumor resistance to anti-PD-1/PD-L1 treatment, which significantly improves the local and distant control of tumors. However, for different metastatic situations, radiotherapy plays different roles in the combination therapy. In oligometastatic status, radiotherapy can be used as a local radical treatment aiming to eliminate cancers in cooperation with systemic PD-1 inhibitors. In other circumstances, like bulky metastasis or multiple metastatic tumors, radiotherapy can be used as adjuvant to systemic immunotherapy. This review focuses on the underlying mechanisms and optimization strategies for the combination of radiotherapy and anti-PD-1/PD-L1 therapy in metastatic disease.

## Introduction

Targeting programmed cell death protein-1(PD-1)/programmed cell death ligand-1 (PD-L1) is one of key achievements in cancer immunotherapy. PD-1/PD-L1 inhibitors have been approved for the treatment of many kinds of tumors, such as melanoma, renal cell carcinoma, lung cancer, esophageal cancer, head and neck cancer, bladder cancer, breast cancer and so on (1). However, the response rate of most tumors treated with PD-1/PD-L1 inhibitors as monotherapy is limited to 15–25% (2). The therapy is even ineffective in some tumors, such as microsatellite stable (MSS) colorectal cancer and pancreas ductal adenocarcinoma (2, 3).Therefore, considerable interest is being directed to use combinational treatments to amplify immunomodulatory effects and produce a synergistic effect to anti-PD-1/PD-L1 therapy (4).

Ionizing radiation can enhance the immune response by directly acting on tumor cell DNA, generating *in situ* tumor vaccine effects, and producing cytokines, which can crosstalk with immune cells, thus changing tumor microenvironment (5). Although “abscopal effect” has been identified more than 67 years, it is very rare to see this phenomenon caused by radiotherapy alone (6). For patients with multiple metastatic tumors, emerging data suggested that single site irradiation was not sufficient enough to boost synergistic effect (7). Over the years, many clinical trials have been launched aiming to examine the safety and efficacy of radiotherapy in combination with immunotherapy. In metastatic cancers, radiotherapy can be used not only as a local radical therapy in some oligometastatic conditions, but also as a sensitizer to PD-1/PD-L1 inhibitors in other circumstances like bulky disease or multiple metastases. However, the optimal radiation doses, fraction size, appropriate timing, irradiated sites, and numbers of irradiated targets have not yet been established. In this study, we mainly discuss the mechanisms and treatment strategies for radiation therapy in combination with PD-1/PD-L1 inhibitors.

## The Potential Mechanisms of Radiation on Immunomodulation

### The Direct Killing Effect of Ionizing Radiation on Tumor Cells

The ionizing radiation affects the tumor cell DNA, causing DNA double-strand breaks and releasing into the cytoplasm (8). Cytoplasmic DNA can activate cyclic GMP-AMP synthase (cGAS) to synthesize cyclic GMP-AMP (cyclic GMP-AMP, cGAMP) and further activate stimulator of interferon genes (STING), which can promote type I interferon (IFN-I) synthesis, thus stimulating dendritic cells (DC) and T cell activation (9). However, the activation of the cGAS/STING pathway is closely related to the radiation dose. Preclinical experiments have shown that hypofractionated (8 Gy×3 fractions) but not ablative radiation (20 Gy single dose) can activate this pathway and induce an abscopal effect when combined with immune checkpoint inhibitors (ICI). When a single dose is 12–18 Gy, the expression of DNA exonuclease Trex1 is significantly increased, resulting in a decrease of cytoplasmic double-stranded DNA, which is not conducive for activating immune response (10).

### Ionizing Radiation Coverts Tumor Into an *in-situ* Vaccine

Radiotherapy is shown to cause tumor cell death associated with releasing tumor-associated antigens (TAAs), danger signals and cytokines which are highly immunogenic and related with initiation of an *in-situ* vaccine (11). The ionizing radiation can promote tumor cells releasing TAAs, especially tumor neoantigens (TNAs), into blood and induce immunogenic cell death (ICD) (12, 13). ICD is a form of regulated cell death that elicits an adaptive immune response and relies upon the antigenicity and adjuvanticity of dying tumor cells (12). TNAs have poor structure homology to self-epitopes and are recognized by self-reactive T cells (12). Accumulating evidence showed the favorable immunotherapy response in patients with high tumor mutation burden (TMB) was in consistent with more TNAs found in this type of cancers (14). Therefore, enhancing tumor antigenicity by inducing TAAs releasing could promote immunogenic response and efficacy of PD-1/PD-L1 treatment (14–16). Radiotherapy can increase the expression of TAAs and release TAAs by causing tumor cell damage, and further promote antigen cross-presentation by DCs and stimulate the activity of antigen-specific cytotoxic CD8^+^T cells, thus eliciting long-term anti-tumor efficacy when combined with PD-1/PD-L1 inhibitors (17).

**Figure 1:**
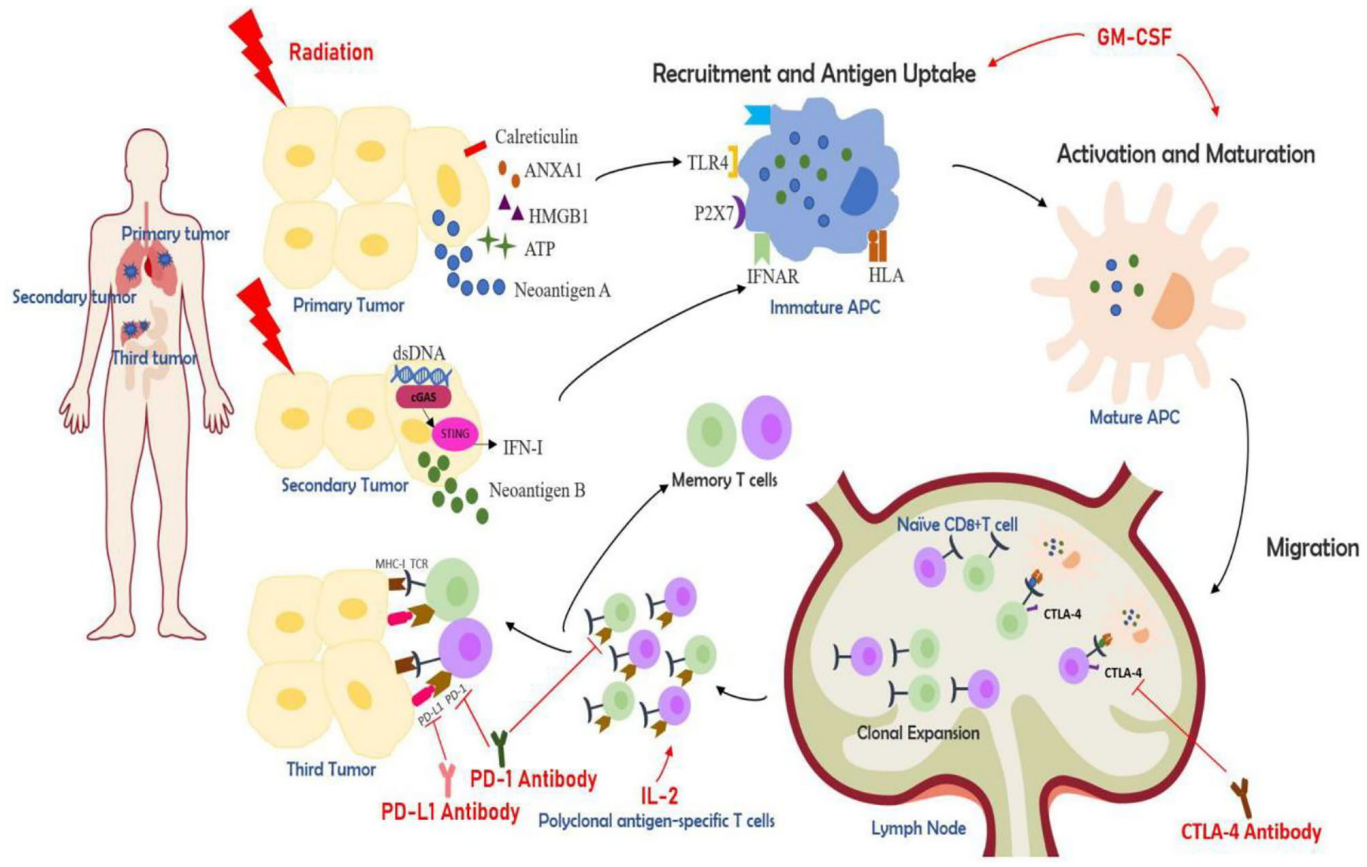
The partial mechanisms of multisite radiotherapy combined with immune checkpoint inhibitors (ICIs) and biological response modifiers (GM-CSF or IL-2). Radiotherapy induces immunogenic cell death (ICD), which exposes and releases danger-associated molecular patterns (DAMPs) like calreticulin, HMGB1, ATP, ANAX1, and similar (12). cGAS-STING pathway is activated by the cytolytic double-strand DNA and results in the release of IFN-I (10). Radiation can also generate tumor neoantigens. Multisite radiotherapy can overcome the insufficient tumor-associated antigen (TAA) exposure caused by tumor heterogeneity (7). ICDs can recruit antigen-presenting cells (APC) like dendritic cells (DC). APCs can take up antigens and further be activated, which can be augmented by GM-CSF. DCs then migrate to lymph nodes, presenting antigens to T cells and prime a cytotoxic T lymphocyte (CTL)-mediated immune activation (26, 27). The activated CTLs initiate clonal proliferation and then travel to the irradiated lesions or distant tumor sites, exerting killing effects. The cytokine IL-2 is essential for the proliferation, differentiation, and survival of T cells (28). CTLA-4 antibody, PD-1 antibody, and PD-L1 antibody, known as ICIs, can increase CTL activation and boost the synergistic anti-tumor effects.

Ionizing radiation can also promote the tumor cells to increase the expression or release of danger-associated molecular patterns (DAMPs) and cytokines which are associated with initiation of adaptive immunity. Several ICD-associated DAMPs and cytokines are found to play important roles in ionizing radiation induced ICD. Calreticulin (CRT) is a ubiquitous calcium-binding protein in the endoplasmic reticulum which can provide DC with a phagocytic signal allowing DC to recognize dead cells and phagocytose (18). Human high mobility group box 1 (HMGB1) is another DAMP that can exert a powerful immunomodulatory effect by binding Toll-like Receptor (TLR)-4 and TLR-9. HMGB1 can further promoting DC maturation and migration to lymph nodes, cross-presenting antigens to naive T cells (19, 20). Adenosine triphosphate (ATP) binds to the purinergic receptor P2X7, which increases the expression of inflammatory cytokines and chemokines, and induces the phagocytosis and inflammasome activation of DC (9, 18). Subsequently activated DC can secrete interleukin (IL)-1β and promote the activation of interferon-gamma-producing CD8^+^T cell (11). Cytokines like IFN-I, which is produced by activated STING/TBK/IRF3/ NF-κB signaling pathway, mediates the anti-tumor effect of DC (9, 18).Tumor cell nucleic acid derivatives and extracellular annexin A1 have important roles in initiating ICD and affect the strength and durability of adaptive anti-tumor immune response (21, 22). Other immunostimulants like heat shock proteins, chemokines also play important roles in priming adaptive immunity (23–25). Herein, ionizing radiation can induce ICD and convert tumors into an *in-situ* personalized vaccine, providing immunostimulatory effects (Figure 1).

### Ionizing Radiation Modulates the Tumor Immune Microenvironment

The presentation and recognition of tumor-associated antigens are very important for initiating adaptive immune response, however, a microenvironment with a high density of tumor-infiltrating lymphocytes (TILs) is also essential for eradicating tumor cells. Smyth et al. suggested the tumor immune microenvironment can be categorized into four types according to the infiltration of CD8^+^T cells and the expression of PD-L1 (29), and in 2019 they reclassified in gene level based on a T cell inflammatory gene signature and TMB (30). Turan et al. suggest that three landscapes best define the cancer microenvironment: immune-active, immune-deserted, and immune-excluded landscape (31). Among them, the tumors with immune desert microenvironment are also called “cold” tumors and generally resistant to ICIs (32). The “immune desert” microenvironment is characterized by the presence of a small amount of TIL and a large number of type II tumor-associated macrophages (TAM), myeloid suppressor cells (MDSC), regulatory T cells (Treg), and other suppressive immune cells (33). Both tumor cells and suppressive immune cells can produce molecules promoting tumor growth, such as vascular endothelial growth factor (VEGF), IL-10, transforming growth factor (TGF)-β, adenosine, and prostaglandin E2. These molecules can prevent DC activation and inhibit the activation of cytotoxic T cells (CTLs) and nature killer (NK) cells (34).

Ionizing radiation can modulate the tumor microenvironment and overcome the barriers of immune suppression. Chemokines like chemokine (C-X-C motif) ligand (CXCL)-9, CXCL-10, CXCL-16 are upregulated after irradiation. These chemokines have an important role in the recruitment of T cells into local tumor microenvironment and activation of T cells (35). Ionizing radiation can also convert TAM into TAM-1, which can secrete inducible nitric oxide synthase (iNOS), upregulate the expression of intercellular adhesion molecule-1 (ICAM-1) and vascular cell adhesion molecules (VCAM) to facilitate lymphocytes infiltrating into tumor tissues (36, 37). Ionizing radiation can directly improve the killing ability of CTLs and NK cells. Tumors inhibit host immune response by downregulating major histocompatibility complex I (MHC-I), a key molecule of CD8^+^T cell recognition, as well as secreting negative immune factors and recruiting immunosuppressive cells (17). However, radiotherapy can increase the expression of MHC-I and II molecules, Fas death receptors and stress ligands on tumor cells surface, which stimulates T cells and NK cells medicated cytotoxicity (38–40). Therefore, ionizing radiation can promote the infiltration of immune cells into the tumor microenvironment and directly improve the recognition and killing ability of T cells and NK cells, which potentially boosting the systemic efficacy of ICIs.

## Exploration the Best Mode of Radiotherapy and PD-1/PD-L1 Inhibitors

Ionizing radiation is a double-edged sword. In addition to immune activation effects, it also has immunosuppressive effect (41). DNA double-strand breaks caused by ionizing radiation can activate ATM/ATR/Chk1 kinase signaling pathway, thereby up-regulating PD-L1 expression and inhibiting T cells activity (42, 43). Ionizing radiation can promote tumor cells to release transforming growth factor-β (TGF-β), IL-33, and other cytokines to increase the recruitment of Tregs (44). CD73 (ecto-5'-nucleotidase), which can be upregulated by ionizing radiation, can generate adenosine and increase Tregs in the tumor microenvironment (45). Tregs can induce effector T cells apoptosis, inactivation, dormancy, and inhibit the functions of B cells, NK cells, DC and macrophages (34). Therefore, it is not only necessary to consider how to exert the optimal immune activation effect of ionizing radiation but also how to avoid immunosuppressive effects when combining with anti-PD-1/PD-L1 therapy.

### Exploration of the Dose and Fraction Size of Radiotherapy

So far, the optimal dose and fraction schedule of radiotherapy to sensitize PD-1/PD-L1 inhibitors has not been determined. Many preclinical studies investigated the potential impacts on the immunity with different radiation doses. Kulzer et al. (46) found that hypofractionated treatment (5 Gy×3 fractions) could enhance tumor necrosis factor (TNF)-α, IL-6, and IL-8 levels comparing to conventional fractionated radiotherapy (2 Gy×5 fractions), suggesting that hypofractionated radiotherapy (HFRT) may promote the maturation and activation of antigen-presenting cells, especially DC. Lan et al. (47) found that HFRT could reduce MDSC infiltration into the tumor microenvironment in mice models. When combined with PD-L1 antibody, a higher tumor control effect was observed in HFRT treated mice comparing to those treated with conventional schemes (47). In fact, radiation doses exceeding 5 Gy per fraction can effectively and directly destroy tumor cells and render these cells' elements for *in-situ* vaccination (5, 20, 48). On the other hand, the conventional schedules are more likely to cause systemic lymphopenia which affects immunotherapy efficacy and associated with poor prognosis (49–51).

However, a higher single dose per fraction is not always associated with a higher immune activation effect. Evidence showed that 7.5–10 Gy×2–3 fractions could stimulate immune response with lower level of Tregs and achieve a better tumor control effect comparing to 15 Gy×1 fraction (52). Studies have also found that >12 Gy irradiation can inhibit the STING pathway and down-regulate IFN-I by up-regulating Trex1, which can decompose cytoplasmic double-stranded DNA. In contrast, the free double-stranded DNA is obviously elevated at a dose of 8–12 Gy, and the STING pathway is activated (10). Filatenkov et al. (53) found that hypofrationated irradiation (15 Gy×2–3 fractions) can reduce MDSCs when compared with a single dose fraction mode (30 Gy×1 fraction), thereby promoting higher activation of T cell function.

Some clinical trials have shown the clinical activity and safety of combination radiotherapy and PD-1/PD-L1 inhibitors in metastatic tumors. In the phase I trial conducted by Luke et al. (54), the 10–15 Gy×3 fractions scheme combined with pembrolizumab showed safe antitumor activity. The overall response rate (ORR) was 13.6% and <10% subjects experienced ≥ grade 3 adverse reactions. A phase II trial, PEMBRO-RT, examined the effect of 8 Gy×3 fractions radiotherapy combined with pembrolizumab in advanced metastatic non-small cell lung cancer (NSCLC). Comparing to the single pembrolizumab treatment without SBRT in control group, SBRT with pembrolizumab showed 36% ORR at 12 weeks (control 18%, *p* = 0.07), median progression free survival (PFS) of 6.6 month (control 1.9 month, *p* = 0.19) and median overall survival (OS) 15.9 month (control 7.6 month, *p* = 0.16) (55). In MDACC trial, where pembrolizumab was concurrently given with SBRT (50 Gy in four fractions) or HFRT (45 Gy in 15 daily fractions) as experimental group, no benefits in median PFS or OS were observed when compared with pembrolizumab without radiation therapy (56). But the pooled analysis of PEMBRO-RT and MDACC trials demonstrated that adding radiotherapy to pembrolizumab provided significant survival benefit (57). Moreover, subgroup analysis showed that 50 Gy in four fractions were significantly associated with better PFS (57), which needs further validation by a randomized phase III trial. The most common adverse events (AEs) in both trials were fatigue, respiratory related symptoms, rash, pruritus and weight loss. Generally, the AEs were mild and self-limiting in patients received pembrolizumab and radiotherapy, comparable with the safety profile in patients received pembrolizumab alone.

Radiotherapy schedules for patients with oligometastasis or multiple metastasis need tailored. The ESTRO/EORTC consensus on oligometastasis recommends combing local radical treatment with systematic treatment to eliminate the disease. Thorough local treatment can reduce the resistance to current systemic treatment and restore sensitivity to systemic therapy by eradicating metastasis (58). In oligometastatic tumors, the SABR-COMET study showed that radical or nearly radical SBRT (30–60 Gy in 3–8 fractions, 16–24 Gy in 1 fraction allowed for intracranial lesions) had significant OS benefits (the 5-year OS rate was 42.3 vs. 17.7%) compared to palliative treatment (8 Gy in 1 fraction or 30 Gy in 10 fractions) in the control group (59, 60). However, the number of patients with grade 2 or higher treatment-related toxicities was increased to 29% following the use of SABR compared with 9% in the control group. Therefore, for patients with multiple metastases, the accessibility and safety of radical treatment must be considered. Palliative radiotherapy may be more suitable for reducing tumor burden and enhancing the sensitivity of systemic therapy. Further research needs to investigate the combination of palliative HFRT and ICIs in patients with multiple metastases in order to determine the optimal dose and fraction size to enhance tumor response to immunotherapy without increasing treatment related toxicity. Meanwhile, radiation therapy schedule can be individualized based on different tumor pathological types, tumor sizes, tumor locations, metastatic states, intrinsic radiosensitivity, and host characteristics (61).

In the trials of oligometastatic disease listed in Tables 1, 2, radiotherapy was administered according to the lesion and clinical condition location, trying to achieve a radical dose [biologically effective dose (BED)>100 Gy] with 8–12 Gy per fraction in most of the trials. The palliative dose schedules of 6–15 Gy×3–5 fractions or a single dose of 20 Gy were given for multiple metastatic cancers. These trials helped us to determine the doses in different tumors and metastatic conditions in the future.

**Table 1:** Trials testing radiotherapy in combination with PD-1/PD-L1 in advanced metastatic cancers that allowed only one irradiated lesion or did not mention the irradiated numbers.

**NCT number**	**Phase**	**Tumor type**	**RT regimen**	**PD-1/PD-L1 inhibitors**	**Treatment schedule timing**	**Trial design (arms)**	**Primary outcome**	**Status**
NCT03988647	II	Metastatic Merkel cell carcinoma	9 Gy × 3f or 4–6 Gy × 5f	Pembrolizumab	RT will be given between the first and second cycles of immunotherapy	Single group	ORR	Recruiting
NCT03220854	II	Advanced solid tumors	6–12 Gy × 3–5f	Humanized anti-PD-1 monoclonal antibody	PD-1 inhibitor will be started after last SRT fraction (on same day)	Single group	Proportion of patients with improved disease control	Active, not recruiting
NCT03548428	II	Oligometastase in Sarcoma	SBRT:3 to 5 fractions depending on tumor size	Atezolizumab	Not mentioned	Arm A: SBRT+Atezolizumab Arm B:SBRT	PFS	Recruiting (62)
NCT02843165	II	Advanced metastatic disease	9.5 Gy × 3 allowed reduction (6 Gy × 3 Minimum Dose)	Anti-CTLA-4 and anti-PD-1/PD-L1 antibodies	SBRT will be delivered within 1–21 days of the start of Cycle 1 of the CBI	Arm A: CBI plus SBRT Arm B: CBI	ORR	Recruiting
NCT04166734	I/II	Advanced malignant pleural mesothelioma	10 Gy × 3f	Pembrolizumab	Pembrolizumab will be given prior to SBRT	Sequential assignment Non-randomized	AE	Not yet recruiting
NCT03436056	I/II	Metastatic NSCLC	10 Gy × 3f 18 Gy × 3f dosed at the maximum tolerated dose	Pembrolizumab	Pembrolizumab will be given prior to SBRT	Sequential assignment Non-randomized	AE.To establish the recommended dose	Active, not recruiting
NCT02992912	II	Metastatic tumors (colorectal cancer, NSCLC, RCC, sarcoma)	15 Gy × 3f	Atezolizumab	Not mentioned	Single Group	PFS	Recruiting
NCT03115801	II	Metastatic genitourinary cancers	10 Gy × 3f	Nivolumab Atezolizumab Pembrolizumab	PD-1/PD-L1 inhibitor is administered on the day of radiation (Day 1)	Arm A:immunotherapy alone Arm B:Radiation and immunotherapy	ORR	Active, not recruiting
NCT02400814	I	Stage IV NSCLC	Total of five fractions	MPDL3280A	Arm A:concurrent Arm B:induction cohort Arm C:sequential cohort	Arm A:SBRT Beginning on day 1 of course 1 Arm B:SBRT Beginning on day 1 of course 3 Arm C:SBRT prior to anti-PD-L1	To determine best administration schedule of MPDL3280A and SBRT	Active, not recruiting
NCT04098432	I/II	Locally advanced unresectable pancreatic adenocarcinoma	8 Gy × 4f	Nivolumab	Nivolumab is given after SBRT	Single Group	AE	Recruiting
NCT03509584	I	Pretreated advanced stage non-small cell lung cancer	8 Gy × 3f	Nivolumab	Not mentioned	Arm I:HFRT+ Nivolumab Arm II: HFRT+ Nivolumab + ipilimumab	AE	Recruiting
NCT04306926	II	Advanced oligometastatic NSCLC	Give according to the location of the lesion and clinical condition.	TQB2450	SBRT 3 days before TQB2450	Single group	PFS	Not yet recruiting
NCT02599779	II	TKI refractory metastatic kidney cancer (mRCC) patients	Dose and duration dependent on body site	Pembrolizumab	Arm-A: SBRT will be given at the time of progression on pembrolizumab and pembrolizumab will be continued. Arm B: SBRT will be given before the 2nd course of pembrolizumab and pembrolizumab will be continued.	Arm A: SBRT will be given at the time of progression on pembrolizumab and pembrolizumab will be continued. Arm B: SBRT will be given before the 2nd course of pembrolizumab and pembrolizumab will be continued.	PFS	Recruiting
NCT04547452	II	Advanced metastatic HCC	7–10 Gy × 5–8f	Sintilimab	The first course of sintilimab will be given within 4–6 weeks after completion of SBRT.	Arm A: Sintilimab and SBRT Arm B:Sintilimab	PFS	Recruiting
NCT03035890	I/II	Metastatic NSCLC	8–15 Gy × 3f 6–10 Gy × 5f	Nivolumab Pembrolizumab Atezolizumab	Concurrent	Single group	ORR	Active, not recruiting
NCT03122496	I	Metastatic anaplastic thyroid cancer	9 Gy × 3 f	Durvalumab	RT is given within 2 weeks after the completion of cycle 1 of durvalumab and tremelimumab	Single group	OS	Active, not recruiting
NCT03867175	III	Metastatic lung cancer	3–10 treatments of SBRT	Pembrolizumab	Not mentioned	Arm A:SBRT and Pembrolizumab Arm B:Pembrolizumab	PFS	Recruiting
NCT02826564	I	Metastatic urothelial cancer	SBRT	Pembrolizumab	Arm A:Sequential Arm B:Concurrent	Arm A:SBRT prior to pembrolizumab Arm B:SBRT concurrent with pembrolizumab	AE DLT	Completed (63)
NCT03101475	II	Colorectal cancer liver metastases	10 Gy × 3 f	Durvalumab	SBRT is started 8–14 days after first dose of immunotherapy	Single group	ORR	Recruiting
NCT04167657	II	Advanced NSCLC	6 Gy × 5f	Sintilimab	Sintilimab is started no later than 3 weeks after radiation.	Single group	ORR	Recruiting
NCT04361162	II	MSS pancreatic cancer	Not mentioned	Nivolumab	Concurrent	Single group	ORR	Recruiting

*We searched “radiation and PD-1/PD-L1 inhibitors” in the clinicaltrials.gov database to identify studies with the following statuses: not yet recruiting, enrolling by invitation, recruiting, active, not recruiting, completed, and unknown status (Clinical Trial). A total of >60 trials were found. We identified the trials using radiotherapy with PD-1/PD-L1 inhibitors in advanced metastatic cancers (date of final query, 25 November 2020). Then we searched “radiation and immunotherapy” in the clinicaltrials.gov database as above. A total of >150 trials were found. We identified the studies of radiotherapy with PD-1/PD-L1 inhibitors in advanced metastatic cancers (date of final query, 25 November 2020). This list shows the trials that allowed only one irradiated lesion or did not mention the irradiated numbers. This list should not be considered comprehensive or exhaustive*.

*SABR, stereotactic ablative radiotherapy; PFS, progression free survival; CBI, checkpoint blockade immunotherapy; ORR, objective response rate; SBRT, stereotactic body radiotherapy; RCC, renal cell carcinoma; AE, adverse events; DLT, dose limiting toxicities; RT, raidotherapy; TKI, tyrosine kinase inhibitor; NSCLC, non-small-cell lung cancer; SCC, squamous cell carcinoma; HNSCC, head and neck squamous cell carcinoma; ICI, immune checkpoint inhibitor; HCC, hepatocellular carcinoma; OS, overall survival; ACC, adenoid cystic carcinoma; MSS, microsatellite stability*.

**Table 2:** Trials testing radiotherapy in combination with PD-1/PD-L1 in advanced metastatic cancers that allowed more than one irradiated lesions.

**NCT number**	**Phase**	**Tumor type**	**RT regimen**	**PD-1/PD-L1 inhibitors**	**Treatment schedule timing**	**Numbers of irradiated targets**	**Trial design (arms)**	**Primary outcome**	**Status**
NCT03464942	II	Advanced triple negative breast cancer	SABR 20 Gy × 1f or 8 Gy × 3f	Atezolizumab	PD-1 inhibitor will be started within 5 days of last SABR dose	1–4 metastases with at least 1 untreated	Arm A:Single Dose Arm B:Fractionated Dose	PFS	Recruiting
NCT03283605	I/II	Metastatic head and neck carcinomas	Not mentioned	Durvalumab Tremelimumab	SBRT will be administered between Cycle 2 and 3 of durvalumab and tremelimumab	2–5	Single group	AE PFS	Recruiting (64)
NCT03644823	II	Advanced NSCLC	6 Gy × 3f	Atezolizumab	Not mentioned	1–2	Single group	AE	Recruiting
NCT03812549	I	Stage IV NSCLC	SBRT 10 Gy × 3f	Sintilimab	Sintilimab will be started within 7 days after radiation completed	At least 2	Single group	AE and/or DLT	Recruiting
NCT04549428	II	Advanced oligoprogressive NSCLC	8 Gy × 1f	Atezolizumab	RT will be delivered concomitant to the 2nd dose of atezolizumab	All eligible metastatic and primary sites	Single group	ORR	Not yet recruiting
NCT04625894	I	Oligometastatic gastrointestinal cancer	Multisite SABR (BED > 100 Gy)	Camrelizumab	SABR prior to PD-1 inhibitor	Multisite	Single group	DLT	Not yet recruiting
NCT02303366	I	Oligometastatic breast neoplasia	20 Gy × 1f	MK-3475	SABT followed by MK-3475	At least one metastases (to a maximum of five metastases)	Single group	Safety profile	Completed
NCT03223155	I	Metastatic lung cancer	Three or five fractions of radiation	Nivolumab	Sequential Arm: nivolumab/ipilimumab between 1 and 7 days after completion of SBRT. Concurrent Arm: nivolumab/ipilimumab first and must complete planned SBRT to 2–4 sites within 2 weeks	2–4	Sequential Arm Concurrent Arm	AE	Recruiting
NCT03087019	II	Recurrent or metastatic ACC	>5 fractions	Pembrolizumab	Concurrent	Up to 5	Arm A: Pembrolizumab + Radiation Arm B: Pembrolizumab	ORR	Active, not recruiting
NCT04535024	II	MSS oligometastatic colorectal cancer	Target dose will be adjusted depending on site of the lesion and organs at risk (BED > 100 Gy).	Sintilimab	Starts within 1 week upon SABR completion	Sequence of irradiation for multiple metastases	Single group	ORR	Recruiting
NCT03825510	II	Metastatic non-small cell lung cancer	3–5 fractions of SBRT	Nivolumab or Pembrolizumab	PD-1 inhibitors start after SBRT	≤3 sites	Single group	OS and acute toxicity	Recruiting
NCT02608385	I	Advanced solid tumors	3 or 5 doses of SBRT	Pembrolizumab	Pembrolizumab is given after SBRT	All sites in Oligometastatic tumors	Arm A: Dose Escalation Cohort. Patients will be enrolled to receive specific doses of SBRT to determine the best safe doses. Arm B: Large Volume Tumors Cohort. Tumors will be partially treated with SBRT. Arm C: Oligometastatic Cohort. All lesions will be treated with SBRT	Recommended SBRT dose in combination with pembrolizumab	Active, not recruiting

*We searched “radiation and PD-1/PD-L1 inhibitors” in the clinicaltrials.gov database to identify studies with the following statuses: not yet recruiting, enrolling by invitation, recruiting, active, not recruiting, completed, and unknown status, with study type of interventional (Clinical Trial). A total of >60 trials were identified as trials using radiotherapy with PD-1/PD-L1 inhibitors in advanced metastatic cancers (date of final query, 25 November 2020). Then we searched “radiation and immunotherapy” in the clinicaltrials.gov database as above. A total of >150 trials were detected. We identified the studies of radiotherapy with PD-1/PD-L1 inhibitors in advanced metastatic cancers (date of final query, 25 November 2020). This list shows the trials that allowed more than one lesion irradiated. This list should not be considered comprehensive or exhaustive*.

*SABR, stereotactic ablative radiotherapy; PFS, progression free survival; CBI, checkpoint blockade immunotherapy; ORR, objective response rate; SBRT, stereotactic body radiotherapy; RCC, renal cell carcinoma; AE, adverse events; DLT, dose limiting toxicities; RT, raidotherapy; TKI, tyrosine kinase inhibitor; NSCLC, non-small-cell lung cancer; SCC, squamous cell carcinoma; HNSCC, head and neck squamous cell carcinoma; ICI, immune checkpoint inhibitor; HCC, hepatocellular carcinoma; OS, overall survival; ACC, adenoid cystic carcinoma; MSS, microsatellite stability*.

### Exploration of the Timing Schedule of Combination Therapy

Selecting an appropriate timing for combining radiotherapy and anti-PD-1/PD-L1 therapy is also crucial when designing the scheme. Preclinical data suggested that the PD-L1 expression significantly increased after irradiation. Higher level of PD-L1 expression was found at a single dose of 10 Gy comparing to 5 Gy, and at 48 h after radiation comparing to at 24 h (36). Dovedi et al. (65) found that highest expression of PD-L1 on tumor cells was at 3 days after radiotherapy, and PD-1 on T cells was upregulated 1–7 days after radiotherapy. *In vivo* preclinical data also suggested that concurrent anti-PD-1/PD-L1 antibodies administration with conventionally fractionated RT had longer survival time than those treated sequentially (65). However, there are other evidences suggested that different timing of radiation therapy and ICI therapy (concurrent or sequentially) can also produce synergistic effects (66–68). Herter-Sprie et al. showed that there was no significant difference among concurrently PD-1 antibody administrated with RT, and sequentially giving RT at 5 or 7 days after PD-1 antibody administration (69). Therefore, from the perspective of preclinical data, there are different results even some contradictions about the timing schedule, and there is still no conclusion of the optimal timing.

Although the optimal timing of combination of RT and ICI is not determined, this combinational therapy shows notable efficiency. In metastatic NSCLC, the experimental group given pembrolizumab within 1 week after SBRT showed better clinical effects compared with pembrolizumab administrated alone in the control group in PEMBRO-RT study (55). A phase I study for solid metastatic tumors showed that sequential administration of pembrolizumab after SBRT at multiple metastatic lesions achieved 13.2% in ORR and 13.5% abscopal effect in non-irradiated metastases (54). Regarding to investigation of best combinational timing, PACIFIC study showed stage III unresectable NSCLC patients who received durvalumab within 14 days after concurrent chemotherapy and radiotherapy (CCRT) had longer PFS than those received durvalumab over 14 days after completion of CCRT (70, 71). Similar result was reported that melanoma patients with brain metastasis who received PD-1 inhibitor and CTLA-4 inhibitor treatment within 4 weeks after stereotactic radiosurgery (SRS) had better results compared to those received PD-1 inhibitor and CTLA-4 inhibitor over 4 weeks after SRS (18). These trials implied that patient receiving PD-1/PD-L1 inhibitors immediately after radiotherapy might have better clinical outcome. However, there were several arguments. A phase I clinical study showed that ORR of simultaneous SBRT treatment after 3 cycles of PD-1 inhibitor was significantly better than that of SBRT followed by PD-1 inhibitor sequential treatment (72). The COSINR phase I trial evaluated concurrent or sequential ipilimumab, nivolumab, and SBRT in patients with stage IV NSCLC and found that the median PFS was 5.9 months in the sequential arm and 6.2 months in the concurrent arm, which showed no significant differences in two different timing schedule (73).

The safety and toxicity of radiotherapy and anti-PD-1/PD-L1 therapy are of great concern. Pembrolizumab given concurrently with SBRT or HFRT confirmed no clinical benefits in the MDACC trial but two patients had grade 4 adverse event which might be related to the concurrent scheme (56). Anti-PD-1/PD-L1 therapy may also lead to radiation recall pneumonitis (74). In the clinical studies listed above, it seemed the time intervals between radiotherapy and anti-PD-1/PD-L1 therapy were not associated with the rate of severe pneumonitis. Nonetheless, a study presented at the ESMO 2020 congress suggested that the application of anti-PD-1 drugs before or during thoracic radiotherapy increases the incidence of radiation pneumonitis compared to administration after radiotherapy (60 vs. 28%, *p* = 0.01) (75). Bang et al. showed higher overall toxicity when radiation was administered within 14 days of immunotherapy (39 vs. 23%, *p* = 0.06) but no significant differences in grade 3 AEs (76). These data seems that concurrent scheme has more adverse reactions and inferior effectiveness than sequential therapy, but it is still controversial due to the lack of randomized controlled trials. However, it is notable that the overall toxicity may also related with high BED, irradiated volumes and irradiated sites (77). Future studies are needed for better understanding of the efficacy and safety of different schedules and defining suitable patients for the options listed in Tables 1, 2.

### Exploration of Appropriate Volume and Numbers of Irradiated Targets in Combination Therapy

In 2019, Chang et al. suggested using multisite radiotherapy for metastatic sites instead of single-site irradiation to boost the synergistic effect (7). Considering the heterogeneity among different metastatic sites, only one lesion irradiation in patients with multiple metastases might not be sufficient to expose new TAAs and promote immune cell infiltration to all metastatic sites. In addition, the increased tumor burden may lead to a decrease in the efficacy of PD-1 inhibitors (11, 78).Therefore, multisite irradiation can obviously decrease tumor burden, and consequently restore the tumors' sensitivity to anti-PD-1/PD-L1 therapy. However, multisite treatment undoubtedly increases the irradiated volume and adverse reactions. Treatment-related lymphopenia was associated with a less effective response to anti-PD-1/PD-L1 therapy and inferior survival (49, 50, 79). Therefore, it may be helpful to maintain the number and function of immune cells so that they can be recruited to initiate anti-tumor immune response. This might be achieved through decreasing the exposure of circulating blood volume and avoiding irradiation at lymphoid tissue or medullary tissue, such as bone marrow, spleen, thymus, and lymphatic vessels (11).

For patients with oligometastatic disease, defined as number of metastases equal or <5 and restricted to no more than 2 organs, several studies have shown that active local treatment for all metastases can significantly prolong patients' OS with tolerable side effects (58–60). The phase II clinical study done by Bauml et al. (80) showed median PFS of 18.7 months (PEMBRO-RT: 6.6 months) and median OS of 41.6 months (PEMBRO-RT: 15.9 months) in patients with oligometastatic (≤4) NSCLC treated with local treatment (surgery, radiotherapy, radiofrequency ablation) combined with a PD-1 inhibitor for all lesions. The results may suggest better survival benefit of radical radiotherapy done for all metastatic sites if applicable than done at only one site. However, benefits of maximizing irradiated sites with concurrent ICI therapy need to be examined in randomized controlled phase III clinical trials.

Multisite SBRT is relatively implementable in patients with oligometastatic disease and small tumor size. However, it is not practical to give all sites SBRT to patients with multiple metastases or bulky tumors. Partial tumor irradiation can be considered in certain conditions with controlled, tolerable toxicity. In the phase I trial mentioned above, patients with solid metastatic tumors administrated with multisite SBRT with pembrolizumab achieved 13.2% in ORR. Partial tumor irradiation was carried out if the target tumor volume was larger than 65 mL in these patients (54). Other partial irradiation strategies like novel SBRT targeted hypoxic segment, called bystander tumor volume (BTV), defined by PET and contrast-enhanced CT, showed very inspiring results suggesting a bulky tumor control rate of 95% (bystander effects) and non-irradiated metastases of 45% (abscopal effects) (81, 82). Other ways like spatially fractionated radiation therapy (SFRT, also known as GRID) can precisely treat target lesion with a non-uniform dose and minimize the toxicity to normal tissue. Preclinical evidence suggested that SFRT could further trigger immune responses and abscopal effects, which might be a potential combination modality with PD-1 inhibitors, especially for bulky tumors (83–85).

The safety and efficacy of multiple cycles of HFRT with each cycle delivering to one lesion instead of one cycle simultaneous multisite radiotherapy combining with anti-PD-1/PD-L1 immunotherapy is tested in our clinical trial (ChiCTR1900026175), presented at the ASCO congress 2020. Participants who had solid tumors with multi-metastases failed to standard therapy were enrolled and treated with PD-1 inhibitors, radiotherapy and GM-CSF (PRaG regimen) sequentially. Three doses of 8 Gy or five doses of 5 Gy are delivered to tumor lesion based on its site and size. On the 2nd day after radiotherapy, PD-1 antibody is intravenously administered once, and GM-CSF 200 μg is subcutaneously injected daily for 2 weeks. At least 2 cycles of triple combination are required, and each cycle is repeated every 3 weeks with different lesions irradiated. After completion of PRaG regimen, maintenance therapy with PD-1 inhibitor is administered every 3 weeks until disease progression or unacceptable toxicity. Interim analysis showed a favorable short-term efficacy of 3-month ORR of 15.8% and PFS of 4.0 months with tolerable toxicity (86, 87). Currently the study is ongoing.

There are other ways to get more lesions irradiated to boost anti-PD-1 effects by combing SBRT with low-dose radiation therapy (LDRT). Welsh et al. proposed to promote immune response to cancer by utilizing high-dose and low-dose radiation synergistically. Clinical data provided a promising result, where 58% of the low dose target responded to a mean dose of 7.3 Gy (1.1–19.4 Gy), which was remarkably higher than no-dose lesions (18%, *p* = 0.0001) (88). The underlying rationale is high-dose radiation increases the release and presentation of antigens as well as activates immunity, while low-dose radiation promotes the infiltration of immune cells into the tumor microenvironment (88). On-going phase I study in metastatic NSCLC reported delivering SBRT in 30 Gy in 3 fractions to a small volume target and LDRT (2 Gy×1 fraction, 4 Gy×2 fractions, or 10 Gy×5 fractions) to a large lesion, with administering sintilimab within 1 week after radiotherapy completion, achieves an ORR of 78.6%. There are 80% of subjects experience grade 1–2 treatment-related adverse events (TRAE) and only 6.7% of subjects have ≥G3 TRAE (89).

It is not clear how many lesions irradiated are required to obtain the greatest immune sensitization effect and minimize side effects for patients with advanced multiple metastatic tumors. At present, there are no large randomized controlled studies. There are several clinical studies on SBRT irradiation of multiple metastases combined with PD-1 inhibitor therapy are underway (Table 2). In addition to investigate the optimal radiotherapy schedule to tumors, the metastatic sites and their biological behaviors should also be considered when selecting the irradiated targets (90). Clinical data showed that radiotherapy targeting to parenchymal sites, such as liver and lung, might cause a better systemic immune changes than targeting to non-parenchymal sites, such as brain and bone (91). In 2018, Pitroda et al. biologically identified three distinct molecular subtypes of colorectal liver metastases, which was related to clinical outcomes and was potential independently of established clinical risk factors (92). These finding suggested that the molecular subtypes of oligometastasis can predict a subset of patients who might benefit most from local treatment (90). Therefore, lesions selected for radiotherapy can not only be considered by numbers and volumes but also be determined according to the molecular characteristics of metastases.

## Biological Response Modifiers to Boost the Effect of Combinational Therapy

The addition of biological immunomodulators can further boost the effect of this combinational approach. Cytokines like IFN-α, IL-2, GM-CSF, TNF-α, IL-15, IL-12, have a synergistic action with radiotherapy (93). In this review, we are mainly focusing on IL-2 and GM-CSF (Figure 1).

The cytokine IL-2 is secreted by effector T cells and is essential for the proliferation, differentiation, and survival of T cells. Preclinical studies have shown that in mouse models of melanoma, colon and breast cancer, HFRT combined with IL-2 can produce significant synergistic therapeutic effects and enhance anti-tumor effects of CD8^+^T cells and NK cells (94). Phase I clinical study showed that in metastatic malignant melanoma and renal cancer, SBRT combined with IL-2 was well-tolerated and provided an ORR of 66.6%. The possible mechanism is the activation of CD4^+^ effector memory T cells by combinational treatment (28). To date, there is no available data in clinical trials for radiotherapy combined with IL-2 and PD-1/PD-L1 inhibitors. A small number of phase I/II clinical studies are currently underway (Table 3).

**Table 3:** Trials testing radiotherapy in combination with PD-1/PD-L1 and cytokines (IL-2 or GM-CSF).

**NCT number**	**Phase**	**Tumor type**	**RT regimen**	**PD-1/PD-L1 inhibitors**	**Treatment schedule timing**	**Primary outcome**	**Status**
NCT03474497	I/II	Metastatic NSCLC, Melanoma, RCC, or HNSCC who have failed PD-1/ PD-L1 inhibitors	8 Gy × 3f	Pembrolizumab	Radiotherapy will be delivered to the treatment lesion during the second cycle of therapy using an 8 Gy × 3 fractions palliative regimen.A total of four interleukin-2 treatments will be delivered into the treatment lesion by IT injection biweekly (at least 48 h apart) starting 24–96 h after the completion of radiotherapy and to be completed during the second on-trial cycle of Pembrolizumab.	Abscopal response rate	Recruiting
NCT03224871	Early Phase I	Metastatic NSCLC	8 Gy × 3f	Nivolumab	Nivolumab will be started on week 1 day 1, concurrent with radiotherapy	DLT	Completed
NCT03958383	I/II	Melanoma	Palliative radiation therapy	Nivolumab	Phase IA: Participants receive hu14.18-IL2 fusion protein IT. Phase IB: Participants undergo palliative RT and hu14.18-IL2 fusion protein IT as in phase IA. Phase IC: Participants undergo palliative RT, receive nivolumab, and hu14.18-IL2 fusion protein IT as in phase IA. Phase ID: Participants undergo palliative RT, receive nivolumab in combination with ipilimumab, and hu14.18-IL2 fusion protein IT as in phase IA.	AE MTD MAD	Recruiting
NCT04106180	II	Advanced NSCLC	8 Gy × 3f	Sintilimab	SBRT combined sintilimab and GM-CSF	ORR	Recruiting
ChiCTR1900026175	I/II	Metastatic solid tumor	8 Gy × 3f	PD-1/PD-L1 inhibitors	SBRT combined PD-1/PD-L1 inhibitors and GM-CSF	Safety PFS Incidence of abscopal effects	Recruiting
ChiCTR2000035817	I/II	Advanced liver cancer	Not mentioned	Carrelizumab	SBRT combined PD-1/PD-L1 inhibitors and GM-CSF	PFS	Recruiting

*We searched “radiation and IL-2” in the clinicaltrials.gov database to identify studies, and 18 trials were found. Data were obtained searching “SBRT and IL-2” in the clinicaltrials.gov database resulting in 4 trials, where 3 trials were on combining radiotherapy with PD-1/PD-L1 inhibitors and IL-2 (date of final query, 25 November 2020). Then we searched “IL-2” in www.chictr.org.cn database to identify studies; 17 trials were identified; no study met our requirements. Then we searched “radiation and GM-CSF” in the clinicaltrials.gov database. Thirty-seven trials were identified. Data were obtained searching “SBRT and IL-2” in the clinicaltrials.gov database to identify studies, and 5 trials were detected. We identified one study on radiotherapy with PD-1/PD-L1 inhibitors in advanced metastatic cancers (date of final query, 25 November 2020). Then we searched “GM-CSF” in www.chictr.org.cn database to identify studies; 10 trials were identified, where 2 studies were on combining radiotherapy with PD-1/PD-L1 inhibitors and GM-CSF. This list should not be considered comprehensive or exhaustive*.

*HNSCC, head and neck squamous cell carcinoma; NSCLC, non-small-cell lung cancer; RCC, renal cell carcinoma; MTD, maximum tolerated dose; MAD, maximum administered dose; AE, adverse events; DLT, dose limiting toxicities; SBRT, stereotactic body radiotherapy; ORR, objective response rate; PFS, progression free survival; IT, intratumorally*.

GM-CSF is also an immunomodulatory cytokine, which can promote the differentiation of monocyte/M1 type macrophages and DCs, enhance their activities and antigen presentation, and amplify the body's immune response (26, 95). Previous studies showed that the expression level of DC gene signature in renal cell carcinoma and NSCLC tissues was positively correlated with OS (27). Blocking PD-L1 on DC can reduce the isolation of PD-L1 from B7.1, thus enhancing the interaction between B7.1/CD28 and activating T cells (27). Animal experiments suggested that GM-CSF combined with ICIs can enhance the activity of innate immune cells by enhancing antigen presentation, indirectly recruiting T cells into the tumor microenvironment, and ultimately enhancing the efficacy of PD-1/PD-L1 inhibitor. Thus, GM-CSF may help to transform “cold” tumors into “hot” tumors (96).

Clinical studies have also demonstrated that GM-CSF can enhance the efficacy of immune checkpoint inhibitors. In a randomized controlled study of patients with unresectable stage III or IV melanoma, the median OS of the patients treated with GM-CSF and ipilimumab was significantly improved compared to the group treated without GM-CSF (97). Preliminary findings in patients with advanced cholangiocarcinoma showed pembrolizumab combined with GM-CSF improved 6 months PFS reached 35% with 7% of subjects having ≥ G3 adverse reactions, suggesting this combination is safe and obtained good short-term effect (98). Evidence from combination PD-1/PD-L1 inhibitors with GM-CSF modified tumor vaccines also demonstrated synergistic anti-tumor effects (99–101). GM-CSF could also boost the immune effect of radiotherapy and induce abscopal effects. Prospective clinical study has shown that local radiotherapy combined with GM-CSF induces a 27% abscopal effect and improves patients' prognosis in patients with advanced solid tumors (102). To date, there is no report on triple combination therapy of radiotherapy, PD-1/PD-L1 blocker and GM-CSF. Our prospective study on HFRT combined with PD-1 blocker and GM-CSF in the treatment of advanced multiple metastatic solid tumors is ongoing (ChiCTR1900026175) (86, 87). Several phase II clinical studies of second-line SBRT combined with PD-1 inhibitors and GM-CSF triple therapy in solid tumors are ongoing (Table 3).

## Summary

Combination treatment of radiotherapy and PD-1/PD-L1 inhibitors is a promising strategy for patients with metastatic cancers, where radiotherapy acts as a radical local treatment in oligometastasis and as an adjuvant therapy in multiple disease or bulky disease by directly damaging malignant cells, helping TAA releasing and antigen presentation, modulating tumor microenvironment. Addition of biological immunomodulators can further amplify the anti-tumor immune effects of this combinational treatment. Further research needs to optimize treatment schedule, maximize immune response and reduce adverse effects, through investigation of doses and fraction size of radiotherapy, the numbers and sites for irradiation, as well as the optimal timing of combination. It will provide solid evidence for this combinational treatment to support it widely accepted in clinical practice in the future.

## Data Availability Statement

The raw data supporting the conclusions of this article will be made available by the authors, without undue reservation.

## Author Contributions

XZ, JP, and ZX helped to write and revise the manuscript. All authors contributed to the article and approved the submitted version.

## Conflict of Interest

ZX was employed by ICON Plc. The remaining authors declare that the research was conducted in the absence of any commercial or financial relationships that could be construed as a potential conflict of interest.

## References

[1] Miller KD Nogueira L Mariotto AB Rowland JH Yabroff KR Alfano CM. Cancer treatment and survivorship statistics, 2019. CA Cancer J Clin. (2019) 69:363–85. 10.3322/caac.21565 31184787

[2] Ribas A Wolchok JD. Cancer immunotherapy using checkpoint blockade. Science. (2018) 359:1350–5. 10.1126/science.aar4060 29567705 PMC7391259

[3] Yamamoto K Venida A Yano J Biancur DE Kakiuchi M Gupta S. Autophagy promotes immune evasion of pancreatic cancer by degrading MHC-I. Nature. (2020) 581:100–5. 10.1038/s41586-020-2229-5 32376951 PMC7296553

[4] Tang J Yu JX Hubbard-Lucey VM Neftelinov ST Hodge JP Lin Y. Trial watch: the clinical trial landscape for PD1/PDL1 immune checkpoint inhibitors. Nat Rev Drug Discov. (2018) 17:854–5. 10.1038/nrd.2018.210 30482962

[5] Bernstein MB Krishnan S Hodge JW Chang JY. Immunotherapy and stereotactic ablative radiotherapy (ISABR): a curative approach? Nat Rev Clin Oncol. (2016) 13:516–24. 10.1038/nrclinonc.2016.30 26951040 PMC6053911

[6] Demaria S Formenti SC. The abscopal effect 67 years later: from a side story to center stage. Br J Radiol. (2020) 93:20200042. 10.1259/bjr.20200042 32101479 PMC7217574

[7] Brooks ED Chang JY. Time to abandon single-site irradiation for inducing abscopal effects. Nat Rev Clin Oncol. (2019) 16:123–35. 10.1038/s41571-018-0119-7 30401936

[8] Bhalla N Brooker R Brada M. Combining immunotherapy and radiotherapy in lung cancer. J Thorac Dis. (2018) 10(Suppl.13):S1447–60. 10.21037/jtd.2018.05.107 29951296 PMC5994496

[9] Weichselbaum RR Liang H Deng L Fu YX. Radiotherapy and immunotherapy: a beneficial liaison? Nat Rev Clin Oncol. (2017) 14:365–79. 10.1038/nrclinonc.2016.211 28094262

[10] Vanpouille-Box C Alard A Aryankalayil MJ Sarfraz Y Diamond JM Schneider RJ. DNA exonuclease Trex1 regulates radiotherapy-induced tumour immunogenicity. Nat Commun. (2017) 8:15618. 10.1038/ncomms15618 28598415 PMC5472757

[11] Golden EB Marciscano AE Formenti SC. Radiation therapy and the *in situ* vaccination approach. Int J Radiat Oncol Biol Phys. (2020) 108:891–8. 10.1016/j.ijrobp.2020.08.023 32800803

[12] Galluzzi L Vitale I Warren S Adjemian S Agostinis P Martinez AB. Consensus guidelines for the definition, detection and interpretation of immunogenic cell death. J Immunother Cancer. (2020) 8:e000337corr1. 10.1136/jitc-2019-000337corr1 32209603 PMC7064135

[13] Mouw KW Goldberg MS Konstantinopoulos PA D'Andrea AD. DNA damage and repair biomarkers of immunotherapy response. Cancer Discov. (2017) 7:675–93. 10.1158/2159-8290.CD-17-0226 28630051 PMC5659200

[14] Rizvi NA Hellmann MD Snyder A Kvistborg P Makarov V Havel JJ. Cancer immunology. Mutational landscape determines sensitivity to PD-1 blockade in non-small cell lung cancer. Science. (2015) 348:124–8. 10.1126/science.aaa1348 25765070 PMC4993154

[15] Lhuillier C Rudqvist NP Elemento O Formenti SC Demaria S. Radiation therapy and anti-tumor immunity: exposing immunogenic mutations to the immune system. Genome Med. (2019) 11:40. 10.1186/s13073-019-0653-7 31221199 PMC6587285

[16] Le DT Durham JN Smith KN Wang H Bartlett BR Aulakh LK. Mismatch repair deficiency predicts response of solid tumors to PD-1 blockade. Science. (2017) 357:409–13. 10.1126/science.aan6733 28596308 PMC5576142

[17] Turgeon GA Weickhardt A Azad AA Solomon B Siva S. Radiotherapy and immunotherapy: a synergistic effect in cancer care. Med J Aust. (2019) 210:47–53. 10.5694/mja2.12046 30636308

[18] Goto T. Radiation as an *in situ* auto-vaccination: current perspectives and challenges. Vaccines. (2019) 7:100. 10.3390/vaccines7030100 PMC6789649 31455032

[19] Sato H Okonogi N Nakano T. Rationale of combination of anti-PD-1/PD-L1 antibody therapy and radiotherapy for cancer treatment. Int J Clin Oncol. (2020) 25:801–9. 10.1007/s10147-020-01666-1 32246277 PMC7192886

[20] Vanpouille-Box C Pilones KA Wennerberg E Formenti SC Demaria S. *In situ* vaccination by radiotherapy to improve responses to anti-CTLA-4 treatment. Vaccine. (2015) 33:7415–22. 10.1016/j.vaccine.2015.05.105 26148880 PMC4684480

[21] Garg AD Vandenberk L Fang S Fasche T Van Eygen S Maes J. Pathogen response-like recruitment and activation of neutrophils by sterile immunogenic dying cells drives neutrophil-mediated residual cell killing. Cell Death Differ. (2017) 24:832–43. 10.1038/cdd.2017.15 28234357 PMC5423108

[22] Vacchelli E Ma Y Baracco EE Sistigu A Enot DP Pietrocola F. Chemotherapy-induced antitumor immunity requires formyl peptide receptor 1. Science. (2015) 350:972–8. 10.1126/science.aad0779 26516201

[23] Ahmed A Tait SWG. Targeting immunogenic cell death in cancer. Mol Oncol. (2020) 14:2994–3006. 10.1002/1878-0261.12851 33179413 PMC7718954

[24] Krombach J Hennel R Brix N Orth M Schoetz U Ernst A. Priming anti-tumor immunity by radiotherapy: dying tumor cell-derived DAMPs trigger endothelial cell activation and recruitment of myeloid cells. Oncoimmunology. (2019) 8:e1523097. 10.1080/2162402X.2018.1523097 30546963 PMC6287777

[25] Fucikova J Kralikova P Fialova A Brtnicky T Rob L Bartunkova J. Human tumor cells killed by anthracyclines induce a tumor-specific immune response. Cancer Res. (2011) 71:4821–33. 10.1158/0008-5472.CAN-11-0950 21602432

[26] Mehta HM Malandra M Corey SJ. G-CSF and GM-CSF in neutropenia. J Immunol. (2015) 195:1341–9. 10.4049/jimmunol.1500861 26254266 PMC4741374

[27] Mayoux M Roller A Pulko V Sammicheli S Chen S Sum E. Dendritic cells dictate responses to PD-L1 blockade cancer immunotherapy. Sci Transl Med. (2020) 12:eaav7431. 10.1126/scitranslmed.aav7431 32161104

[28] Seung SK Curti BD Crittenden M Walker E Coffey T Siebert JC. Phase 1 study of stereotactic body radiotherapy and interleukin-2–tumor and immunological responses. Sci Transl Med. (2012) 4:137ra74. 10.1126/scitranslmed.3003649 22674552

[29] Teng MW Ngiow SF Ribas A Smyth MJ. Classifying cancers based on T-cell infiltration and PD-L1. Cancer Res. (2015) 75:2139–45. 10.1158/0008-5472.CAN-15-0255 25977340 PMC4452411

[30] O'Donnell JS Teng MWL Smyth MJ. Cancer immunoediting and resistance to T cell-based immunotherapy. Nat Rev Clin Oncol. (2019) 16:151–67. 10.1038/s41571-018-0142-8 30523282

[31] Turan T Kannan D Patel M Matthew Barnes J Tanlimco SG Lu R. Immune oncology, immune responsiveness and the theory of everything. J Immunother Cancer. (2018) 6:50. 10.1186/s40425-018-0355-5 29871670 PMC5989400

[32] Demaria S Coleman CN Formenti SC. Radiotherapy: changing the game in immunotherapy. Trends Cancer. (2016) 2:286–94. 10.1016/j.trecan.2016.05.002 27774519 PMC5070800

[33] Chen DS Mellman I. Elements of cancer immunity and the cancer-immune set point. Nature. (2017) 541:321–30. 10.1038/nature21349 28102259

[34] Menon H Ramapriyan R Cushman TR Verma V Kim HH Schoenhals JE. Role of radiation therapy in modulation of the tumor stroma and microenvironment. Front Immunol. (2019) 10:193. 10.3389/fimmu.2019.00193 30828330 PMC6384252

[35] Matsumura S Demaria S. Up-regulation of the pro-inflammatory chemokine CXCL16 is a common response of tumor cells to ionizing radiation. Radiat Res. (2010) 173:418–25. 10.1667/RR1860.1 20334513 PMC2857712

[36] Rompre-Brodeur A Shinde-Jadhav S Ayoub M Piccirillo CA Seuntjens J Brimo F. PD-1/PD-L1 immune checkpoint inhibition with radiation in bladder cancer: *in situ* and abscopal effects. Mol Cancer Ther. (2020) 19:211–20. 10.1158/1535-7163.MCT-18-0986 31534011

[37] Dillon MT Bergerhoff KF Pedersen M Whittock H Crespo-Rodriguez E Patin EC. ATR inhibition potentiates the radiation-induced inflammatory tumor microenvironment. Clin Cancer Res. (2019) 25:3392–403. 10.1158/1078-0432.CCR-18-1821 30770349 PMC6551222

[38] Thangamathesvaran L Shah R Verma R Mahmoud O. Immune checkpoint inhibitors and radiotherapy-concept and review of current literature. Ann Transl Med. (2018) 6:155. 10.21037/atm.2018.03.09 29862244 PMC5952022

[39] Wang X Schoenhals JE Valdecanas DR Li A Ye H Zhang F. Suppression of major histocompatibility complex (MHC) class i and ii mediates resistance to anti-PD-1 in lung adenocarcinoma tumors that can be overcome by radiationtherapy. Int J Radiat Oncol Biol Phys. (2016) 96:s89. 10.1016/j.ijrobp.2016.06.224

[40] Wan S Pestka S Jubin RG Lyu YL Tsai YC Liu LF. Chemotherapeutics and radiation stimulate MHC class I expression through elevated interferon-beta signaling in breast cancer cells. PLoS ONE. (2012) 7:e32542. 10.1371/journal.pone.0032542 22396773 PMC3291570

[41] Formenti SC Demaria S. Future of radiation and immunotherapy. Int J Radiat Oncol Biol Phys. (2020) 108:3–5. 10.1016/j.ijrobp.2020.04.034 32819614

[42] Deng L Liang H Burnette B Beckett M Darga T Weichselbaum RR. Irradiation and anti-PD-L1 treatment synergistically promote antitumor immunity in mice. J Clin Invest. (2014) 124:687–95. 10.1172/JCI67313 24382348 PMC3904601

[43] Sato H Niimi A Yasuhara T Permata TBM Hagiwara Y Isono M. DNA double-strand break repair pathway regulates PD-L1 expression in cancer cells. Nat Commun. (2017) 8:1751. 10.1038/s41467-017-01883-9 29170499 PMC5701012

[44] Tanaka A Sakaguchi S. Regulatory T cells in cancer immunotherapy. Cell Res. (2017) 27:109–18. 10.1038/cr.2016.151 27995907 PMC5223231

[45] Coleman CN Eke I Makinde AY Chopra S Demaria S Formenti SC. Radiation-induced adaptive response: new potential for cancer treatment. Clin Cancer Res. (2020) 26:5781–90. 10.1158/1078-0432.CCR-20-0572 32554542 PMC7669567

[46] Kulzer L Rubner Y Deloch L Allgäuer A Frey B Fietkau R. Norm- and hypo-fractionated radiotherapy is capable of activating human dendritic cells. J Immunotoxicol. (2014) 11:328–36. 10.3109/1547691X.2014.880533 24512329

[47] Lan J Li R Yin LM Deng L Gui J Chen BQ. Targeting myeloid-derived suppressor cells and programmed death ligand 1 confers therapeutic advantage of ablative hypofractionated radiation therapy compared with conventional fractionated radiation therapy. Int J Radiat Oncol Biol Phys. (2018) 101:74–87. 10.1016/j.ijrobp.2018.01.071 29619980

[48] Formenti SC Demaria S. Radiation therapy to convert the tumor into an *in situ* vaccine. Int J Radiat Oncol Biol Phys. (2012) 84:879–80. 10.1016/j.ijrobp.2012.06.020 23078897 PMC3811126

[49] Chen D Verma V Patel RR Barsoumian HB Cortez MA Welsh JW. Absolute lymphocyte count predicts abscopal responses and outcomes in patients receiving combined immunotherapy and radiotherapy: a prospective-retrospective analysis of 3 phase I/II Trials. Int J Radiat Oncol Biol Phys. (2020) 108:196–203. 10.1016/j.ijrobp.2020.01.032 32036004

[50] Pike LRG Bang A Mahal BA Taylor A Krishnan M Spektor A. The impact of radiation therapy on lymphocyte count and survival in metastatic cancer patients receiving PD-1 immune checkpoint inhibitors. Int J Radiat Oncol Biol Phys. (2019) 103:142–51. 10.1016/j.ijrobp.2018.09.010 30227198

[51] Crocenzi T Cottam B Newell P Wolf RF Hansen PD Hammill C. A hypofractionated radiation regimen avoids the lymphopenia associated with neoadjuvant chemoradiation therapy of borderline resectable and locally advanced pancreatic adenocarcinoma. J Immunother Cancer. (2016) 4:45. 10.1186/s40425-016-0149-6 27532020 PMC4986363

[52] Schaue D Ratikan JA Iwamoto KS McBride WH. Maximizing tumor immunity with fractionated radiation. Int J Radiat Oncol Biol Phys. (2012) 83:1306–10. 10.1016/j.ijrobp.2011.09.049 22208977 PMC3337972

[53] Filatenkov A Baker J Mueller AM Kenkel J Ahn GO Dutt S. Ablative tumor radiation can change the tumor immune cell microenvironment to induce durable complete remissions. Clin Cancer Res. (2015) 21:3727–39. 10.1158/1078-0432.CCR-14-2824 25869387 PMC4537844

[54] Luke JJ Lemons JM Karrison TG Pitroda SP Melotek JM Zha Y. Safety and clinical activity of pembrolizumab and multisite stereotactic body radiotherapy in patients with advanced solid tumors. J Clin Oncol. (2018) 36:1611–8. 10.1200/JCO.2017.76.2229 29437535 PMC5978468

[55] Theelen W Peulen HMU Lalezari F van der Noort V de Vries JF Aerts J. Effect of pembrolizumab after stereotactic body radiotherapy vs pembrolizumab alone on tumor response in patients with advanced non-small cell lung cancer: results of the PEMBRO-RT phase 2 randomized clinical trial. JAMA Oncol. (2019) 5:1276–82. 10.1001/jamaoncol.2019.1478 31294749 PMC6624814

[56] Welsh J Menon H Chen D Verma V Tang C Altan M. Pembrolizumab with or without radiation therapy for metastatic non-small cell lung cancer: a randomized phase I/II trial. J Immunother Cancer. (2020) 8:e001001. 10.1136/jitc-2020-001001 33051340 PMC7555111

[57] Theelen W Chen D Verma V Hobbs BP Peulen HMU Aerts J. Pembrolizumab with or without radiotherapy for metastatic non-small-cell lung cancer: a pooled analysis of two randomised trials. Lancet Respir Med. (2020) 2020:S2213-60030391-X. 10.1016/S2213-2600(20)30391-X 33096027

[58] Guckenberger M Lievens Y Bouma AB Collette L Dekker A deSouza NM. Characterisation and classification of oligometastatic disease: a European Society for Radiotherapy and Oncology and European Organisation for Research and Treatment of Cancer consensus recommendation. Lancet Oncol. (2020) 21:e18-e28. 10.1016/S1470-2045(19)30718-1 31908301

[59] Palma DA Olson R Harrow S Gaede S Louie AV Haasbeek C. Stereotactic ablative radiotherapy for the comprehensive treatment of oligometastatic cancers: long-term results of the SABR-COMET phase II randomized trial. J Clin Oncol. (2020) 38:2830–8. 10.1101/2020.03.26.20044305 32484754 PMC7460150

[60] Palma DA Olson R Harrow S Gaede S Louie AV Haasbeek C. Stereotactic ablative radiotherapy versus standard of care palliative treatment in patients with oligometastatic cancers (SABR-COMET): a randomised, phase 2, open-label trial. Lancet. (2019) 393:2051–8. 10.1016/S0140-6736(18)32487-5 30982687

[61] Chen Y Gao M Huang Z Yu J Meng X. SBRT combined with PD-1/PD-L1 inhibitors in NSCLC treatment: a focus on the mechanisms, advances, and future challenges. J Hematol Oncol. (2020) 13:105. 10.1186/s13045-020-00940-z 32723363 PMC7390199

[62] le Guevelou J Debaigt C Saada-Bouzid E Viotti J Khalladi N Thibouw D. Phase II study of concomitant radiotherapy with atezolizumab in oligometastatic soft tissue sarcomas: STEREOSARC trial protocol. BMJ Open. (2020) 10:e038391. 10.1136/bmjopen-2020-038391 32967883 PMC7513631

[63] Sundahl N De Wolf K Rottey S Decaestecker K De Maeseneer D Meireson A. A phase I/II trial of fixed-dose stereotactic body radiotherapy with sequential or concurrent pembrolizumab in metastatic urothelial carcinoma: evaluation of safety and clinical and immunologic response. J Transl Med. (2017) 15:150. 10.1186/s12967-017-1251-3 28662677 PMC5492401

[64] Bahig H Aubin F Stagg J Gologan O Ballivy O Bissada E. Phase I/II trial of Durvalumab plus Tremelimumab and stereotactic body radiotherapy for metastatic head and neck carcinoma. BMC Cancer. (2019) 19:68. 10.1186/s12885-019-5266-4 30642290 PMC6332607

[65] Dovedi SJ Adlard AL Lipowska-Bhalla G McKenna C Jones S Cheadle EJ. Acquired resistance to fractionated radiotherapy can be overcome by concurrent PD-L1 blockade. Cancer Res. (2014) 74:5458–68. 10.1158/0008-5472.CAN-14-1258 25274032

[66] Rodriguez-Ruiz ME Rodriguez I Garasa S Barbes B Solorzano JL Perez-Gracia JL. Abscopal effects of radiotherapy are enhanced by combined immunostimulatory mabs and are dependent on CD8 T cells and crosspriming. Cancer Res. (2016) 76:5994–6005. 10.1158/0008-5472.CAN-16-0549 27550452

[67] Sharabi AB Nirschl CJ Kochel CM Nirschl TR Francica BJ Velarde E. Stereotactic radiation therapy augments antigen-specific PD-1-mediated antitumor immune responses *via* cross-presentation of tumor antigen. Cancer Immunol Res. (2015) 3:345–55. 10.1158/2326-6066.CIR-14-0196 25527358 PMC4390444

[68] Park SS Dong H Liu X Harrington SM Krco CJ Grams MP. PD-1 restrains radiotherapy-induced abscopal effect. Cancer Immunol Res. (2015) 3:610–9. 10.1158/2326-6066.CIR-14-0138 25701325 PMC4827718

[69] Herter-Sprie GS Koyama S Korideck H Hai J Deng J Li YY. Synergy of radiotherapy and PD-1 blockade in Kras-mutant lung cancer. JCI Insight. (2016) 1:e87415. 10.1172/jci.insight.87415 27699275 PMC5033933

[70] Gray JE Villegas A Daniel D Vicente D Murakami S Hui R. Three-year overall survival with durvalumab after chemoradiotherapy in stage III NSCLC-update from PACIFIC. J Thorac Oncol. (2020) 15:288–93. 10.1016/j.jtho.2019.10.002 31622733 PMC7244187

[71] Antonia SJ Villegas A Daniel D Vicente D Murakami S Hui R. Overall survival with durvalumab after chemoradiotherapy in stage III NSCLC. N Engl J Med. (2018) 379:2342–50. 10.1056/NEJMoa1809697 30280658

[72] Sundahl N Vandekerkhove G Decaestecker K Meireson A De Visschere P Fonteyne V. Randomized phase 1 trial of pembrolizumab with sequential versus concomitant stereotactic body radiotherapy in metastatic urothelial carcinoma. Eur Urol. (2019) 75:707–11. 10.1016/j.eururo.2019.01.009 30665814

[73] Chmura SJ Bestvina CM Karrison TG Jelinek M Juloori A Pointer KB. Safety and efficacy of a randomized phase I trial to evaluate concurrent or sequential ipilimumab, nivolumab, and stereotactic body radiotherapy in patients with stage IV non-small cell lung cancer (COSINR study). Int J Radiat Oncol Biol Phys. (2020) 108(3Suppl.):S72. 10.1016/j.ijrobp.2020.07.2214

[74] Teng F Li M Yu J. Radiation recall pneumonitis induced by PD-1/PD-L1 blockades: mechanisms and therapeutic implications. BMC Med. (2020) 18:275. 10.1186/s12916-020-01718-3 32943072 PMC7499987

[75] Zhang N Zhu X Kong C Song X Chen C Jiang N. Application of anti-PD1 drugs before or during thoracic radiotherapy increases the incidence of radiation pneumonia compared to the application after radiotherapy. Ann Oncol. (2020) 31:S1081. 10.1016/j.annonc.2020.08.1450

[76] Bang A Wilhite TJ Pike LRG Cagney DN Aizer AA Taylor A. Multicenter evaluation of the tolerability of combined treatment with PD-1 and CTLA-4 immune checkpoint inhibitors and palliative radiation therapy. Int J Radiat Oncol Biol Phys. (2017) 98:344–51. 10.1016/j.ijrobp.2017.02.003 28463153

[77] Chicas-Sett R Morales-Orue I Castilla-Martinez J Zafra-Martin J Kannemann A Blanco J. Stereotactic ablative radiotherapy combined with immune checkpoint inhibitors reboots the immune response assisted by immunotherapy in metastatic lung cancer: a systematic review. Int J Mol Sci. (2019) 20:2173. 10.3390/ijms20092173 31052488 PMC6540197

[78] Huang AC Postow MA Orlowski RJ Mick R Bengsch B Manne S. T-cell invigoration to tumour burden ratio associated with anti-PD-1 response. Nature. (2017) 545:60–5. 10.1038/nature22079 28397821 PMC5554367

[79] Ellsworth SG. Field size effects on the risk and severity of treatment-induced lymphopenia in patients undergoing radiation therapy for solid tumors. Adv Radiat Oncol. (2018) 3:512–9. 10.1016/j.adro.2018.08.014 30370350 PMC6200885

[80] Bauml JM Mick R Ciunci C Aggarwal C Davis C Evans T. Pembrolizumab after completion of locally ablative therapy for oligometastatic non-small cell lung cancer: a phase 2 trial. JAMA Oncol. (2019) 5:1283–90. 10.1001/jamaoncol.2019.1449 31294762 PMC6624820

[81] Tubin S Popper HH Brcic L. Novel stereotactic body radiation therapy (SBRT)-based partial tumor irradiation targeting hypoxic segment of bulky tumors (SBRT-PATHY): improvement of the radiotherapy outcome by exploiting the bystander and abscopal effects. Radiat Oncol. (2019) 14:21. 10.1186/s13014-019-1227-y 30696472 PMC6352381

[82] Tubin S Khan MK Salerno G Mourad WF Yan W Jeremic B. Mono-institutional phase 2 study of innovative Stereotactic Body RadioTherapy targeting PArtial Tumor HYpoxic (SBRT-PATHY) clonogenic cells in unresectable bulky non-small cell lung cancer: profound non-targeted effects by sparing peri-tumoral immune microenvironment. Radiat Oncol. (2019) 14:212. 10.1186/s13014-019-1410-1 31771654 PMC6878646

[83] Yan W Khan MK Wu X Simone CB 2nd Fan J Gressen E. Spatially fractionated radiation therapy: history, present and the future. Clin Transl Radiat Oncol. (2020) 20:30–8. 10.1016/j.ctro.2019.10.004 31768424 PMC6872856

[84] Billena C Khan AJ. A current review of spatial fractionation: back to the future? Int J Radiat Oncol Biol Phys. (2019) 104:177–87. 10.1016/j.ijrobp.2019.01.073 30684666 PMC7443362

[85] Kanagavelu S Gupta S Wu X Philip S Wattenberg MM Hodge JW. *In vivo* effects of lattice radiation therapy on local and distant lung cancer: potential role of immunomodulation. Radiat Res. (2014) 182:149–62. 10.1667/RR3819.1 25036982 PMC7670883

[86] Kong Y Zhao X Li Z Xing P Ma Y Tian Y. PD-1 inhibitor combined with radiotherapy and GM-CSF as salvage therapy in patients with chemotherapy-refractory metastatic solid tumors. J Clin Oncol. (2020) 38(15suppl.):e15173. 10.1200/JCO.2020.38.15_suppl.e15173

[87] Kong Y Zhao X Li Z Xing P Ma Y Tian Y. A phase II trial of multisite stereotactic radiation therapy (SRT) or stereotactic body radiation therapy (SBRT) with PD-1 inhibitor and GM-CSF for the treatment of chemo-refractory metastatic solid tumors. Int J Radiat Oncol Biol Phys. (2020) 108(3Suppl.):e176–7. 10.1016/j.ijrobp.2020.07.138233014817

[88] Menon H Chen D Ramapriyan R Verma V Barsoumian HB Cushman TR. Influence of low-dose radiation on abscopal responses in patients receiving high-dose radiation and immunotherapy. J Immunother Cancer. (2019) 7:237. 10.1186/s40425-019-0718-6 31484556 PMC6727581

[89] Yin L Xue J Li R Zhou L Deng L Chen L. Effect of low-dose radiation therapy on abscopal responses to hypofractionated radiation therapy and anti-PD1 in mice and patients with non-small cell lung cancer. Int J Radiat Oncol Biol Phys. (2020) 108:212–24. 10.1016/j.ijrobp.2020.05.002 32417411

[90] Pitroda SP Chmura SJ Weichselbaum RR. Integration of radiotherapy and immunotherapy for treatment of oligometastases. Lancet Oncol. (2019) 20:e434–42. 10.1016/S1470-2045(19)30157-3 31364595

[91] McGee HM Daly ME Azghadi S Stewart SL Oesterich L Schlom J. Stereotactic ablative radiation therapy induces systemic differences in peripheral blood immunophenotype dependent on irradiated site. Int J Radiat Oncol Biol Phys. (2018) 101:1259–70. 10.1016/j.ijrobp.2018.04.038 29891204 PMC6364565

[92] Pitroda SP Khodarev NN Huang L Uppal A Wightman SC Ganai S. Integrated molecular subtyping defines a curable oligometastatic state in colorectal liver metastasis. Nat Commun. (2018) 9:1793. 10.1038/s41467-018-07303-w 29728604 PMC5935683

[93] Palata O Hradilova Podzimkova N Nedvedova E Umprecht A Sadilkova L Palova Jelinkova L. Radiotherapy in combination with cytokine treatment. Front Oncol. (2019) 9:367. 10.3389/fonc.2019.00367 31179236 PMC6538686

[94] Jing H Hettich M Gaedicke S Firat E Bartholoma M Niedermann G. Combination treatment with hypofractionated radiotherapy plus IL-2/anti-IL-2 complexes and its theranostic evaluation. J Immunother Cancer. (2019) 7:55. 10.1186/s40425-019-0537-9 30808414 PMC6390578

[95] Lotfi N Zhang GX Esmaeil N Rostami A. Evaluation of the effect of GM-CSF blocking on the phenotype and function of human monocytes. Sci Rep. (2020) 10:1567. 10.1038/s41598-020-58131-2 32005854 PMC6994676

[96] Gurbatri CR Lia I Vincent R Coker C Castro S Treuting PM. Engineered probiotics for local tumor delivery of checkpoint blockade nanobodies. Sci Transl Med. (2020) 12:eaax0876. 10.1126/scitranslmed.aax0876 32051224 PMC7685004

[97] Hodi FS Lee S McDermott DF Rao UN Butterfield LH Tarhini AA. Ipilimumab plus sargramostim vs. ipilimumab alone for treatment of metastatic melanoma: a randomized clinical trial. JAMA Oncol. (2014) 312:1744–53. 10.1001/jama.2014.13943 25369488 PMC4336189

[98] Kelley RK Mitchell E Behr S Hwang J Fong L. Phase 2 trial of pembrolizumab (PEM) plus granulocyte macrophage colony stimulating factor (GM-CSF) in advanced biliary cancers (ABC): Clinical outcomes and biomarker analyses. J Clin Oncol. (2018) 36:4087. 10.1200/JCO.2018.36.15_suppl.4087

[99] Zhang X Chen H Li G Zhou X Shi Y Zou F. Increased Tim-3 expression on TILs during treatment with the Anchored GM-CSF vaccine and anti-PD-1 antibodies is inversely correlated with response in prostate cancer. J Cancer. (2020) 11:648–56. 10.7150/jca.29705 31942188 PMC6959042

[100] Shi X Zhang X Li J Zhao H Mo L Shi X. PD-1/PD-L1 blockade enhances the efficacy of SA-GM-CSF surface-modified tumor vaccine in prostate cancer. Cancer Lett. (2017) 406:27–35. 10.1016/j.canlet.2017.07.029 28797844

[101] Tian H Shi G Wang Q Li Y Yang Q Li C. A novel cancer vaccine with the ability to simultaneously produce anti-PD-1 antibody and GM-CSF in cancer cells and enhance Th1-biased antitumor immunity. Signal Transduct Target Ther. (2016) 1:16025. 10.1038/sigtrans.2016.25 29263903 PMC5661645

[102] Golden EB Chhabra A Chachoua A Adams S Donach M Fenton-Kerimian M. Local radiotherapy and granulocyte-macrophage colony-stimulating factor to generate abscopal responses in patients with metastatic solid tumours: a proof-of-principle trial. Lancet Oncol. (2015) 16:795–803. 10.1016/S1470-2045(15)00054-6 26095785

